# Modifiable risk factors for dementia and dementia risk profiling. A user manual for Brain Health Services—part 2 of 6

**DOI:** 10.1186/s13195-021-00895-4

**Published:** 2021-10-11

**Authors:** Janice M. Ranson, Timothy Rittman, Shabina Hayat, Carol Brayne, Frank Jessen, Kaj Blennow, Cornelia van Duijn, Frederik Barkhof, Eugene Tang, Catherine J. Mummery, Blossom C. M. Stephan, Daniele Altomare, Giovanni B. Frisoni, Federica Ribaldi, José Luis Molinuevo, Philip Scheltens, David J. Llewellyn, on behalf of the European Task Force for Brain Health Services, Marc Abramowicz, Daniele Altomare, Frederik Barkhof, Marcelo Berthier, Melanie Bieler, Kaj Blennow, Carol Brayne, Andrea Brioschi, Emmanuel Carrera, Gael Chételat, Chantal Csajka, Jean-François Demonet, Alessandra Dodich, Bruno Dubois, Giovanni B. Frisoni, Valentina Garibotto, Jean Georges, Samia Hurst, Frank Jessen, Miia Kivipelto, David J. Llewellyn, Laura McWhirter, Richard Milne, Carolina Minguillón, Carlo Miniussi, José Luis Molinuevo, Peter M. Nilsson, Janice M. Ranson, Federica Ribaldi, Craig Ritchie, Philip Scheltens, Alina Solomon, Wiesje van der Flier, Cornelia van Duijn, Bruno Vellas, Leonie Visser

**Affiliations:** 1 College of Medicine and Health, University of Exeter, Exeter, UK; 2 Deep Dementia Phenotyping (DEMON) Network, Exeter, UK; 3 Department of Clinical Neurosciences, University of Cambridge, Cambridge, UK; 4 Department of Public Health and Primary Care, Cambridge Public Health, University of Cambridge, Cambridge, UK; 5 Department of Psychiatry and Psychotherapy, Medical Faculty, University of Cologne, Cologne, Germany; 6 Department of Psychiatry and Neurochemistry, Institute of Neuroscience & Physiology, the Sahlgrenska Academy at the University of Gothenburg, Mölndal, Sweden; Clinical Neurochemistry Laboratory, Sahlgrenska University Hospital, Mölndal, Sweden; 7 Nuffield Department of Population Health, University of Oxford, Oxford, UK; 8 Centre for Medical Image Computing, Department of Medical Physics and Biomedical Engineering, University College London, London, UK; 9 Department of Radiology and Nuclear Medicine, Amsterdam University Medical Centers, Amsterdam, The Netherlands; 10 Population Health Sciences Institute, Newcastle University, Newcastle upon Tyne, UK; 11 Dementia Research Centre, Institute of Neurology, University College London, and National Hospital for Neurology and Neurosurgery, University College London Hospital, London, UK; 12 Institute of Mental Health, Division of Psychiatry and Applied Psychology, School of Medicine, Nottingham University, Nottingham, UK; 13 Laboratory of Neuroimaging of Aging (LANVIE), University of Geneva, Geneva, Switzerland; 14 Memory Clinic, Geneva University Hospitals, Geneva, Switzerland; 15 Laboratory of Alzheimer’s Neuroimaging and Epidemiology (LANE), Saint John of God Clinical Research Centre, Brescia, Italy; 16 Department of Molecular and Translational Medicine, University of Brescia, Brescia, Italy; 17 Barcelonaβeta Brain Research Center (BBRC), Pasqual Maragall Foundation, Barcelona, Spain; 18 Alzheimer Center Amsterdam, Department of Neurology, Amsterdam Neuroscience, Vrije Universiteit Amsterdam, Amsterdam UMC, Amsterdam, The Netherlands; 19 Life Science Partners, Amsterdam, The Netherlands; 20 Alan Turing Institute, London, UK; 21 2.04 College House, St Luke’s Campus, University of Exeter Medical School, Exeter, EX1 2 LU UK

**Keywords:** Alzheimer’s disease, Dementia, Aging, Brain health services, Risk factors, Risk profiling, Prevention, Public health

## Abstract

We envisage the development of new Brain Health Services to achieve primary and secondary dementia prevention. These services will complement existing memory clinics by targeting cognitively unimpaired individuals, where the focus is on risk profiling and personalized risk reduction interventions rather than diagnosing and treating late-stage disease. In this article, we review key potentially modifiable risk factors and genetic risk factors and discuss assessment of risk factors as well as additional fluid and imaging biomarkers that may enhance risk profiling. We then outline multidomain measures and risk profiling and provide practical guidelines for Brain Health Services, with consideration of outstanding uncertainties and challenges. Users of Brain Health Services should undergo risk profiling tailored to their age, level of risk, and availability of local resources. Initial risk assessment should incorporate a multidomain risk profiling measure. For users aged 39–64, we recommend the Cardiovascular Risk Factors, Aging, and Incidence of Dementia (CAIDE) Dementia Risk Score, whereas for users aged 65 and older, we recommend the Brief Dementia Screening Indicator (BDSI) and the Australian National University Alzheimer’s Disease Risk Index (ANU-ADRI). The initial assessment should also include potentially modifiable risk factors including sociodemographic, lifestyle, and health factors. If resources allow, *apolipoprotein E ɛ4* status testing and structural magnetic resonance imaging should be conducted. If this initial assessment indicates a low dementia risk, then low intensity interventions can be implemented. If the user has a high dementia risk, additional investigations should be considered if local resources allow. Common variant polygenic risk of late-onset AD can be tested in middle-aged or older adults. Rare variants should only be investigated in users with a family history of early-onset dementia in a first degree relative. Advanced imaging with 18-fluorodeoxyglucose positron emission tomography (FDG-PET) or amyloid PET may be informative in high risk users to clarify the nature and burden of their underlying pathologies. Cerebrospinal fluid biomarkers are not recommended for this setting, and blood-based biomarkers need further validation before clinical use. As new technologies become available, advances in artificial intelligence are likely to improve our ability to combine diverse data to further enhance risk profiling. Ultimately, Brain Health Services have the potential to reduce the future burden of dementia through risk profiling, risk communication, personalized risk reduction, and cognitive enhancement interventions.

## Background

Frisoni and colleagues [1] recently highlighted the rationale for primary and secondary dementia prevention and the need for new services aimed at cognitively unimpaired individuals. Primary dementia prevention strategies for individuals with unknown disease markers include modifiable risk factors, lifestyle, and multiple domain interventions to reduce disease incidence. Secondary prevention targets high risk cognitively unimpaired individuals with biomarker evidence of disease pathology, to prevent or delay symptom onset.

Current memory clinics are ill-equipped to deal with the number of cognitively unimpaired individuals seeking help in memory clinics who believe they may be at increased risk of dementia ([2], *this issue*). For this reason, we envision the development of new Brain Health Services (BHSs) with specific missions including dementia risk profiling, risk communication ([3], *this issue*), risk reduction ([4], *this issue*), and cognitive enhancement ([5], *this issue*). Admittedly, BHSs pose specific societal challenges ([6], *this issue*).

This review focuses on the first principle of risk profiling and is the second part of a Special Issue series of six articles, published in *Alzheimer’s Research & Therapy*, which together provide a user manual for BHSs. We provide an overview of modifiable and genetic risk factors, before discussing best practices for the assessment of risk factors in a BHS setting. We also consider the potential assessment of fluid and imaging biomarkers for risk profiling. We then outline multiple domain measures and risk profiling in the context of primary and secondary prevention services. Finally, we provide practical guidelines for BHSs, and consider possible uncertainties, inconsistencies, and challenges.

## Risk factors

### Overview of modifiable risk factors

The concept of prevention being better than cure underpins the growing interest in the role of modifiable risk factors for cognitive impairment and dementia [7]. The 2020 Report of the *Lancet* Commission identified 12 modifiable risk factors, which, with appropriate interventions, could prevent up to 40% of dementia cases worldwide [8]. This may particularly benefit low- and middle-income countries where the prevalence of dementia is thought to be rising faster than in higher income countries [8].

Education is an early life potentially modifiable risk factor linked to late-life dementia risk [8], either by exerting a direct effect on brain structure by, for example, improving vascularization contributing to cognitive reserve, or by shaping healthier behaviors that reduce cardiovascular and cerebrovascular damage [9]. If causality is assumed and low levels of education were eliminated, then it has been estimated this would lead to a 7% reduction in dementia prevalence [8].

Hearing loss, traumatic brain injury (TBI), hypertension, alcohol consumption (> 21 units per week), and obesity have been identified as key potentially modifiable midlife dementia risk factors [8]. Poor encoding of sound may affect brain structure and function, impose higher cognitive load, and reduce social interaction [10]. Oxidative stress, inflammatory effects, and reduced cerebral flow contribute to brain pathology associated with factors such as TBI and hypertension [11]. The percentage reduction in dementia prevalence if these risk factors were eliminated ranges from 8% for hearing loss to 1% for alcohol and obesity [8].

Diabetes, smoking, air pollution, depression, social isolation, and physical inactivity have been identified as potentially modifiable late life dementia risk factors [8]. Diabetes [12] and physical inactivity [13] are associated with an adverse vascular profile, which itself is associated with an increased dementia risk [13]. Diabetes increases inflammation and oxidative stress on the brain [14]. Smoking and air pollution enhance reactive oxygen and inflammatory responses [11, 15]. Depression and social isolation are associated with accelerated brain and cardiovascular aging [16] and poor health behaviors [17]. The reduction in dementia prevalence associated with elimination of these risk factors ranges from 5% for smoking to 1% for diabetes [8].

As our understanding of modifiable risk factors improves, this list of “key” risk factors will need to be updated. Guidelines for risk reduction of cognitive decline and dementia published by the World Health Organization (WHO) had a greater focus on interventions and present building evidence for additional modifiable risk factors such as diet [18]. For example, emerging evidence suggests that diet, cognitive stimulation, vitamin D, and pesticide exposure may also be important [19, 20]. Based upon current evidence, the 12 potentially modifiable dementia risk factors identified in the 2020 Report of the *Lancet* Commission should be incorporated into BHS assessments and prioritized in personalized interventions.

### Genetic risk factors and interactions with modifiable risk factors

**Fig. 1:**
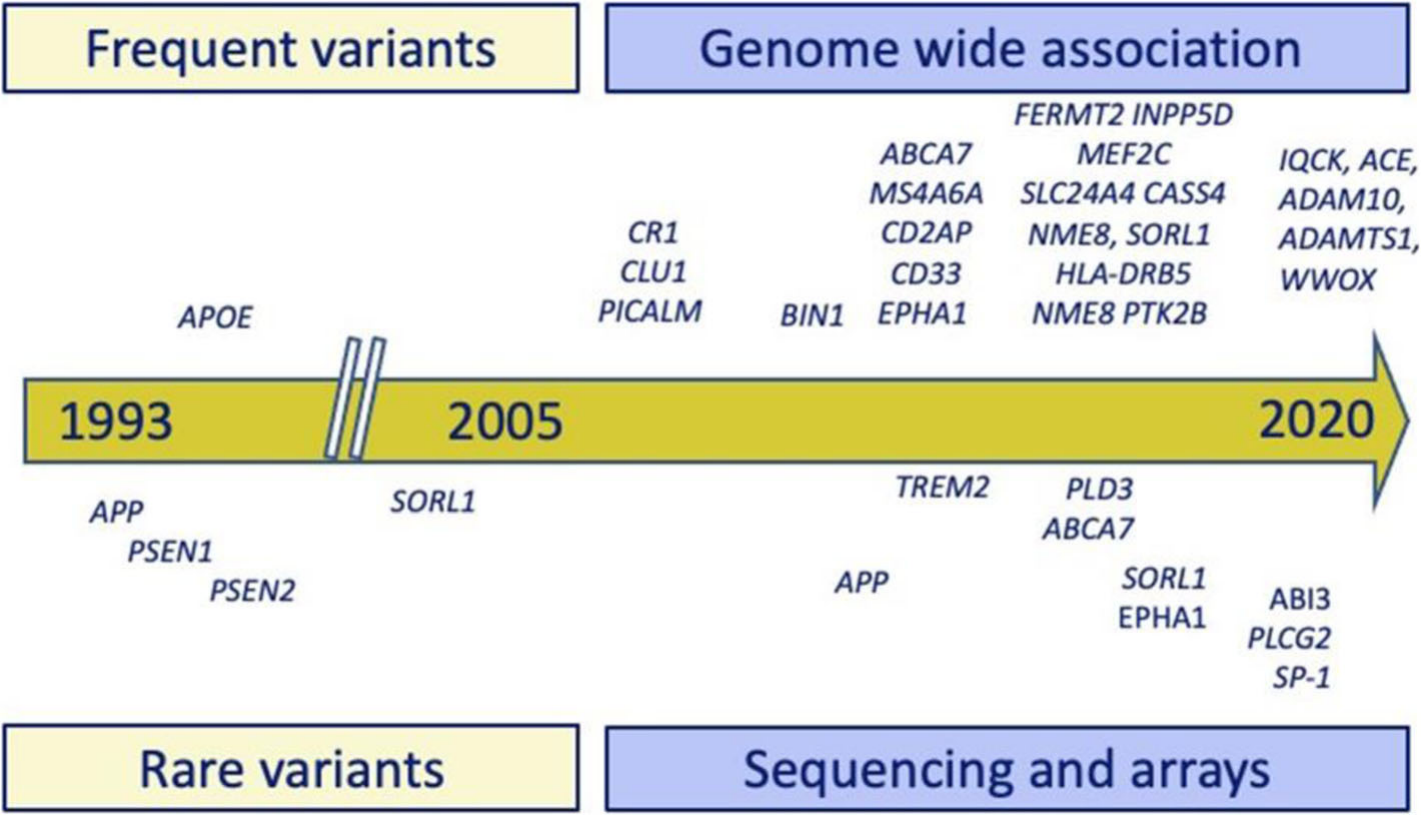
Timeline of the discovery of frequent and rare variants in Alzheimer’s disease. Frequent/common variants discovered in GWAS are shown above the horizontal date axis, and rare variants discovered using sequencing and arrays are shown below the date axis

AD is the most common type of dementia and has a strong genetic component, involving both common and rare genetic variants [21] as illustrated in the discovery timeline summarized in Fig. 1. To date, 34 genetic variants have been associated with AD [22]. Specifically, *PSEN1*, *PSEN2*, and *APP* mutations cause AD dementia in virtually 100% of carriers (autosomal dominant AD [23]), with a mean age at dementia onset of 35–65 years [24] and symptom duration of about 10 years [25]. Nevertheless, the major driver of AD in the general population is the common apolipoprotein E ɛ4 variant (*APOE* ɛ4), which is associated with an elevated risk of developing dementia, i.e., 51–95% in *APOE* ɛ4/ɛ4 and 22–90% in *APOE* ɛ4/- [26, 27] and a mean age at dementia onset of 73–74 years in *APOE* ɛ4/ɛ4 and 75–82 in *APOE* ɛ4/- [28, 29]. Although common variants often have very small effects on a person’s AD risk, jointly they may modify the risk and age at onset of Alzheimer's disease and dementia significantly [22].

Our knowledge of the genes implicated in non-AD dementias is less comprehensive. Mutations in microtubule associated protein tau gene and the gene encoding progranulin are specific for frontotemporal dementia. Other variants including the intronic expansion of a hexanucleotide repeat in C9orf72 and SERPINA1 are also implicated, though few common variants have been identified (see Fig. 1). For dementia with Lewy bodies, only *APOE*, Glucocerebrosidase, and Synuclein Alpha have been replicated. Lastly, for vascular dementia there are no consistent findings for common variants.

It is unknown whether genetic risk modifies the influence of life-style on dementia. Four large multidomain trials of dementia prevention have been conducted. However, only the Finnish Geriatric Intervention Study to Prevent Cognitive Impairment and Disability found a significant difference in the primary outcome following a lifestyle, metabolic, and vascular intervention [30]. The beneficial effect was only observed in *APOE ɛ4* carriers [31]. Null group level findings in other trials may therefore mask effects in genetic subgroups. Comparable findings from observational studies are mixed. There was no interaction between lifestyle factors and polygenic risk score in relation to all-cause dementia risk in the UK Biobank [32]. However, there was a significant interaction between a composite of lifestyle and health factors and APOE/polygenic risk in relation to all-cause dementia in the Rotterdam Study [33]. Taken together, these findings provide suggestive rather than conclusive evidence that, contrary to expectation, those with a high genetic risk may be more likely to benefit from targeted dementia prevention interventions.

## Assessments in the clinic

### Assessing risk factors in the clinic

When assessing cognitively unimpaired users in BHSs, consideration of their modifiable lifestyle and clinical risk factors and their genetic profile can inform personalized and targeted dementia prevention interventions.

#### Assessing potentially modifiable risk factors

The potentially modifiable risk factors to be assessed in BHSs are shown in Table 1, along with examples of methods to assess these factors in a clinical setting.

**Table 1:** Assessment of potentially modifiable risk factors in Brain Health Services

Risk factor	Assessment methods
Education	- International Standard Classification of Education (*applicable across educational systems*) [34] - Years of education (*simple to calculate*) [34]
Lifetime traumatic brain injury	- Ohio State University Traumatic Brain Injury Identification Method (*ideal*) [35] - Medical history or informant or self-reported reports (*practical*)
Hypertension	- Ambulatory devices (*ideal*) - Domestic device (*practical*) Defined as in-office measures at 140/90 and lower in ambulatory or home-based assessments [36].
Alcohol consumption	- Quantity-frequency measures with beverage-specific assessment of time frames and binge-drinking episodes [37] (*ideal*) - > 21 units per week to define high risk (*more practical*)
Obesity and visceral adipose tissue	- Waist circumference (*ideal*) - Body mass index (*practical*) *Note*: There are different ways to measure waist circumference and different cut-offs depending on ethnicity and world region [38].
Hearing impairment	- Pure tone audiometry [39] (*gold standard*) - Whispered Voice Test (*simple but less reliable*) - Speech-in-noise paradigms (*simple but less reliable*) - Questionnaires (*less reliable*)
Diabetes	- Fasting plasma glucose levels (> = 7.0 mmol/l) or HbA1c (> = 6.5%) - Oral glucose tolerance test to diagnose impaired glucose tolerance [40].
Smoking	- Pack years (number of daily packs multiplied by number of years smoking) - Current smoking status (current versus former/never smoker)
Air pollution	- Further research is needed to establish a practical clinically relevant measure.
Depression	- Depression screening measures, e.g., Patient-Health-Questionnaire (PHQ) [41].
Social isolation	- Short questionnaires, e.g., the Lubben Social Network Scale [42] or the Duke Social Support Index [43].
Physical inactivity	- Accelerometers [44] - Heart rate counters [44] - Smart phone or smart watch apps [44] - Self-reported measures (*more practical for clinical setting*)

#### Assessing genetic risk factors

Combining effects of APOE*4 and common variants allows a precise prediction of the risk and age of onset of AD [22, 45, 46] and pathology in the brain [47]. Age specific risk curves may have clinical utility in BHS, allowing determination of future risk of AD at different stages of the life course [22]. These estimates can be provided using polygenic risk scores based on replicated variants [22] assessed by dedicated AD chips or putative genome wide variants that are marginally associated to the disease that can be assessed by general genetics arrays [45–47]. Although in many countries testing for rare variants conferring a high risk of AD (see Fig. 1) is the domain of clinical genetics, routine testing for such variants in a BHS may be useful for users with a family history of AD. Within a BHS, testing for known major genes that harbor rare variants may be done in collaboration with clinical geneticists. As is the case with many complex disorders, rare variants in genes will be encountered for which the functional effects and the risk of AD is yet unknown in archives such as Omim and ClinVar [48]. However, collaboration between the genomics and clinical community could facilitate genetic counseling in the setting of a BHS [48].

### Assessing additional biomarkers

Fluid and neuroimaging biomarkers can be used to differentiate between asymptomatic individuals with and without underlying pathology. This can be used to target people who are particularly likely to benefit from interventions. Assessment of pathology also provides a baseline for disease burden that can be subsequently used to assess rates of progression. Nevertheless, the use of biomarkers in BHSs depends on local facilities and resources and on the context of BHS implementation ([2], *this issue*).

#### Assessing fluid (CSF and plasma) biomarkers

Many studies have consistently shown that core AD cerebrospinal fluid (CSF) biomarkers amyloid β (Aβ_42_ and Aβ_42_/Aβ_40_ ratio), total-tau (T-tau), and phosphorylated tau (P-tau) reflect key elements of AD pathophysiology and have high diagnostic value and high concordance with amyloid positron emission tomography (PET) [49]. However, there are currently no disease specific fluid biomarkers for non-AD dementia. Furthermore, a lumbar puncture is often regarded as complicated and invasive and subjects may have side-effects in the form of transient headache. Thus, for a BHS clinical setting, blood biomarkers are likely to be more practical and acceptable to users than CSF biomarkers.

Technical developments have allowed for quantification of brain-specific proteins in blood samples. For amyloidosis, the plasma Aβ_42_/Aβ_40_ ratio shows high concordance with amyloid PET [50], and can be measured on fully automatized instruments [51]. Blood biomarkers for tau pathology include P-tau181, which shows a marked increase in AD and high concordance with tau PET [52, 53], while levels are normal in other tauopathies, such as frontotemporal dementia. Importantly, plasma P-tau181 is increased in unimpaired elderly having brain amyloidosis, but still a negative tau PET scan [54]. This suggests it may be sensitive to pathological change at an earlier stage. Studies of other tau variants, specifically P-tau217, show encouraging results [55]. Neurofilament light (NFL) is a well-validated neurodegeneration biomarker showing increases in several neurodegenerative disorders, including AD [56], and predicts future rate of cognitive decline [57]. Importantly, plasma NFL increases early in the preclinical phase of AD [58, 59]. While blood biomarkers are very promising, they need further real-world validation before they can be recommended for use in BHSs [60, 61].

#### Assessing imaging biomarkers

Magnetic resonance imaging (MRI) promises to be a sensitive early biomarker of neurodegeneration given that genetic cohorts of dementia demonstrate structural MRI changes many years before symptom onset [62–64]. The finding of selective early hippocampal atrophy is well established in AD [64, 65] and is an accepted biomarker for clinical trials [66, 67], yet translating this into detecting early AD for clinical use requires further work [68]. However, there are a number of potential methodological developments in artificial intelligence, PET, and MRI technology that may lead to more specific and biologically relevant neuroimaging biomarkers [69].

Cerebrovascular risk is a particular focus for neuroimaging studies and impacts on cognition in healthy aging [70]. While silent territorial infarcts are relatively rare, cerebral small vessel disease is extremely common, encompassing white matter hyperintensities, lacunes, widened Virchow-Robin spaces, and cerebral microbleeds [71]. White matter hyperintensities are a frequent finding associated with cardiovascular risk factors such as hypertension and smoking. They significantly increase the risk of stroke, dementia, and overall mortality [72], especially when lesions become confluent [73]. Stroke itself is strongly associated with incident all-cause dementia [74]. Lacunes are found more frequently in individuals with atrial fibrillation and present an independent risk factor for cognitive decline. Cerebral microbleeds can be due to cardiovascular risk factors deep in the basal nuclei, while lobar cerebral microbleeds are reflective of amyloid-angiopathy; they only carry a weak risk for dementia on a population level [75].

Current consensus practice for assessing MRI scans is to use visual rating scales, such as the medial temporal lobe atrophy scale [76], the parietal atrophy scale [77], the global cortical atrophy scale [78], the age-related white matter changes [79], and the Fazekas scale for white matter lesions [80, 81]. Measurement of regional cortical thickness can also identify presymptomatic amyloid positive individuals [82]. However, with the advent of artificial intelligence, new neuroimaging tools for diagnosis and prognosis are emerging [83–85] that may provide more sensitive assessments in the near future.

PET has provided a suite of tools for assessing people with cognitive impairment using specific ligands that bind to physiological targets. The most well established in clinical practice is 18-Fluorodeoxyglucose (FDG) PET which has proved useful for predicting cognitive impairment in Parkinson’s disease [86]. Ligands for beta-amyloid have found the presence of beta-amyloid increases with age, reaching 65% in health over 80s [87]. However, a positive beta-amyloid PET did not correlate to cognition, so the implications of this finding remains uncertain for predicting risk. It has been shown in genetic forms of AD that amyloid accumulates 15–20 years prior to symptom onset and it is thought to be an early critical factor in disease, although changes in amyloid load do not reliably correlate with cognitive change [62]. Other ligands for tau [88] inflammation [89] or synaptic integrity [90] exist, but remain in the research domain. The cost and availability of PET imaging may limit its applicability to BHSs but could have a role in selected high risk individuals.

## Risk profiling

### Multidomain measures and risk profiling

A number of dementia risk prediction models have been developed to determine dementia risk in middle-aged or older adults [91, 92]. The validity of most risk models is unknown, as is the degree to which they can be appropriately used in different populations. Prediction models which have been validated in multiple samples include the Cardiovascular Risk Factors, Aging and Dementia (CAIDE) score [93], the Australian National University Alzheimer’s Disease Risk Index (ANU-ADRI) [94, 95], and the Brief Dementia Screening Indicator (BDSI) [96]. Basic characteristics of these models are shown in Table 2. The CAIDE score assesses long-term risk of dementia in middle-aged adults, whereas the ANU-ADRI and the BDSI predict medium-term AD and dementia risk respectively in older adults. The overall accuracy of these risk prediction models is moderate (range 0.64–0.78), indicating that, although they can be improved upon, they can also generate useful predictions. It is notable that 10 of the 12 modifiable risk factors for dementia included in the 2020 Report of the *Lancet* Commission are included in these models [8]. The only modifiable risk factors identified in that report which are not currently included are hearing loss and air pollution.

**Table 2:** Comparison of selected dementia risk models

	Cardiovascular Risk Factors, Aging and Dementia (CAIDE) score	Australian National University Alzheimer’s Disease Risk Index (ANU-ADRI)	Brief Dementia Screening Indicator (BDSI)
Development sample age range	39–64	Variable (population based)	65+
Development sample size	1409	903–2496	1125–13889
Mean length of follow-up, years	21	Variable (population based)	6
Accuracy (area under the curve or C-statistic)**	0.77–0.78	0.64–0.74	0.68–0.78
*Demographic predictors*			
Age	**●**	**●**	**●**
Sex	**●**	**●**	
Education*	**●**	**●**	**●**
*Functional impairment*			
Difficulty with instrumental activities of daily living			**●**
*Health predictors*			
Systolic blood pressure*	**●**		
Body mass index*	**●**		**●**
Total cholesterol	**●**		
Diabetes*		**●**	**●**
Stroke			**●**
Traumatic brain injury*		**●**	
Depression*/depressive symptoms		**●**	**●**
*Lifestyle predictors*			
Smoking*		**●**	
Physical activity*	**●**	**●**	
Social isolation*		**●**	
Cognitively stimulating activities		**●**	
Alcohol*		**●**	
Fish intake		**●**	
*Genetic predictors*			
*APOE* e4 carrier	**●**		

*Modifiable risk factor identified in the 2020 Report of the *Lancet* Commission [8]

**Range includes the development and validation test results

The CAIDE score has a moderate level of discriminative accuracy over 20 years follow-up (area under the curve (AUC) = 0.77, 95% CI = 0.71–0.83). The addition of APOE e4 did not substantially increase accuracy (AUC = 0.78, 95% CI = 0.72–0.84). When this model was externally validated, it performed similarly in a midlife population (AUC = 0.75) [97] but poorly in late-life populations with shorter follow-up times (AUC range 0.49–0.57) [95]. When tested in three cohorts, the ANU-ADRI was found to have moderate levels of discriminative accuracy for Alzheimer’s disease: Rush Memory and Aging Project study AUC = 0.64 (95% CI = 0.60–0.68), Kungsholmen Project study AUC = 0.74 (95% CI 0.71–0.77), and Cardiovascular Health Cognition study AUC = 0.73 (95% CI = 0.69–0.78). The BDSI was tested in four cohorts including the Cardiovascular Health Study (CHS), The Framingham Heart Study (FHS), the Health and Retirement Study (HRS), and the Sacramento Area Latino Study on Aging (SALSA). The discrimination accuracy of the final model was moderate across cohorts: CHS AUC = 0.68 (95% CI = 0.65–0.72), FHS AUC = 0.77 (95% CI = 0.73–0.82), HRS AUC = 0.76 (95% CI = 0.74–0.77), and SALSA AUC = 0.78 (95% CI = 0.72–0.83).

There have also been attempts to develop new models in at-risk subpopulations. For example, the Diabetes-Specific Dementia Risk Score (DSDRS) is a model for type 2 diabetics. The DSDRS was found to have reasonable accuracy in the development (AUC = 0.74) and validation (AUC = 0.75) cohorts [97]. Disease-specific predictive models may be important as generic dementia risk prediction models may not work well in specific subpopulations [98]. Further, not all prediction models for dementia developed in high-income countries are necessarily applicable to low- and middle-income countries [99].

## Discussion

### Summary

We now have a reasonable idea what the “key” potentially modifiable dementia risk factors are in early-, mid-, and late- life. It is also likely that further risk factors will be added to this list in the future. Of course, optimal risk profiling for identification of patients at high risk of dementia may not necessarily be synonymous with optimal *modifiable* risk factor profiling for targeted interventions. For risk profiling, rare genetic variants for early-onset dementia have been identified, and common variants for late-onset dementia, particularly AD, are now known. Further research is needed to investigate possible gene-environment interactions. CSF biomarkers are not very practical in the context of BHSs; however, blood-based biomarkers may be useful subject to further real-world validation. Structural MRI is becoming established as a clinically useful imaging biomarker of dementia pathologies, and advanced imaging may be a useful supplement to this if available. Existing dementia risk prediction models offer a practical way of risk profiling individual users, though there is room for improvement and they have not yet been optimized for use in BHSs.

### Practical guidelines

**Fig. 2:**
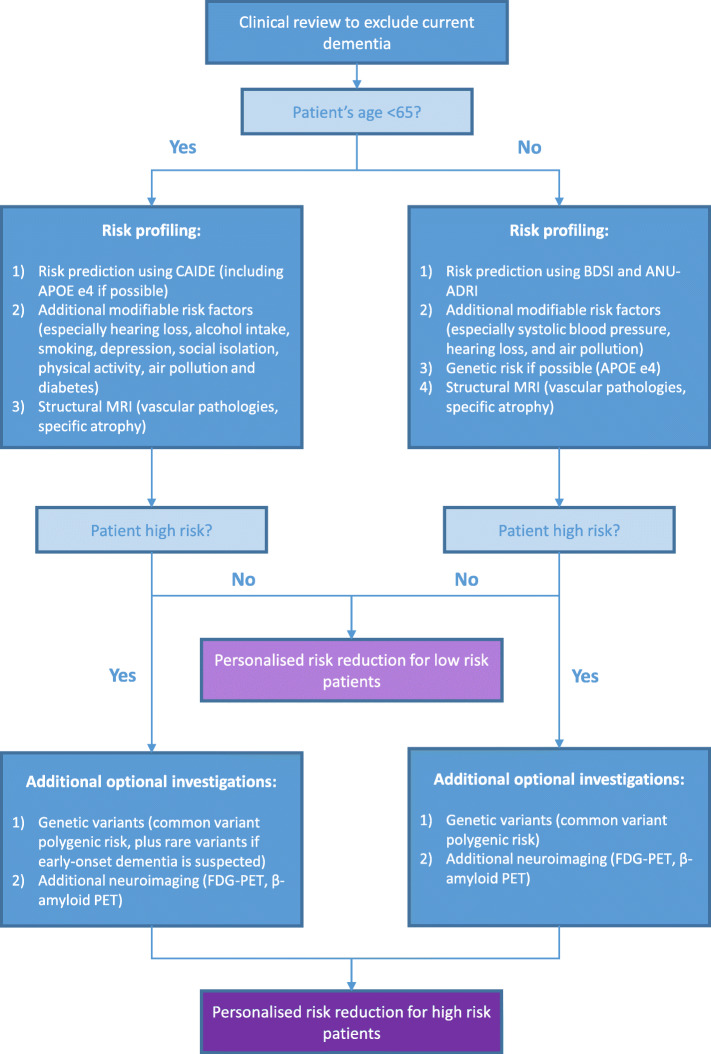
Proposed workflow for dementia risk profiling in BHSs

The assessment of risk factors and risk profiling in BHSs will require a multidisciplinary team, and a balance between precision and practicality. Individual assessment of modifiable risk factors is likely to involve multiple measures and may prove to be time consuming. Some assessments may be completed in advance of the appointment and a specialist nurse appointment may also enrich the information available. The individual will undergo an assessment tailored to their age, level of risk following an initial assessment, and local resources available (see Fig. 2). Comprehensive recommendations for BHSs, including risk profiling outlined here, will be presented in a separate article. A follow-up communication of the user’s results will be required ([3], *this issue*), followed by the proposal of an individualized plan for risk reduction ([4], *this issue*) and/or cognitive enhancement interventions ([5], *this issue*) and/or clinical trial opportunities.

The initial assessment should include the exclusion of pre-existing dementia. Risk profiling should incorporate a multidomain risk profiling measure validated for use with the relevant age group, assessment of additional risk factors, *APOE* ɛ4 status if possible, and structural MRI. To assess long-term dementia risk in middle-aged individuals aged 39–64, we recommend that BHSs use the CAIDE score. The CAIDE should not be used for anyone younger than 39 whose dementia risk will be negligible over 20 years or in those aged 65 years or older as accuracy is poor in older adults and better alternatives are available. *APOE* ɛ4 genotyping should be undertaken if possible to permit use of the full CAIDE model and as multimodal interventions may be more effective in e4 carriers [31]. This will allow for targeted allocation of limited resources when attempting dementia prevention. To assess medium-term dementia risk in individuals aged 65 and older, we recommend that BHSs use the BDSI and the ANU-ADRI which produces a comparable risk prediction for AD specifically. The ANU-ADRI also has the practical advantage of incorporating a larger number of modifiable risk factors such as smoking and physical activity which can inform targeted interventions. Additional risk factor assessment should focus on those factors with the strongest evidence base and greatest opportunity to intervene, particularly those outlined in the 2020 Report of the *Lancet* Commission [8]. These can be divided into early life (education), midlife (hearing loss, TBI, hypertension, alcohol consumption, and obesity), and late-life (diabetes, smoking, air pollution, depression, social isolation and physical inactivity) and should be assessed routinely as appropriate for the user’s age. Some, but not all, of these risk factors are incorporated into existing dementia risk scores (see Table 2). Structural MRI should be used in BHSs to enable the assessment of non-degenerative pathologies (e.g., inflammation, tumors), cerebrovascular burden (particularly cerebral small vessel disease including white matter hyperintensities and lacunes), and neurodegenerative processes (generalized, medial temporal lobe and hippocampal atrophy). Determination of whether a user has high dementia risk may be made on the basis of clinical judgment of the initial assessment results or by formally combining the information using a computerized decision support system, if available.

If initial BHS dementia risk profiling indicates that the user has a low risk of dementia, then low intensity interventions can be implemented, for example signposting to relevant risk reduction public health information and resources or low-cost non-specialist guidance. If however the initial risk profiling indicates that the user has a high risk of dementia then additional optional investigations should be considered if local facilities and resources allow. Common variant polygenic risk of late-onset AD can be tested in middle-aged or older adults. However, rare variants should only be investigated in users with a family history of early-onset dementia (< 65 years) in a first-degree relative in collaboration with clinical geneticists. Advanced imaging with FDG-PET or amyloid PET may also prove to be informative in high risk patients to clarify their burden of underlying pathology. Plasma biomarkers may prove to be a useful additional optional investigation in high risk users in the near future, though they are not currently recommended for use in BHSs before further real-world validation. Future risk profiling tools may therefore benefit from incorporating richer genetic information using polygenic risk scores and more advanced biomarker and imaging findings.

## Conclusions

Risk profiling in BHS involves a core assessment comprised of multidomain risk prediction models in combination with additional risk factors, *APOE* ɛ4 status if possible, and structural MRI. If resources allow, then additional investigations including more comprehensive genetic testing and advanced neuroimaging can be undertaken in high risk users. Further research is necessary to refine risk profiling and risk reduction approaches in low- and middle-income countries. Results can then be communicated to users, a personalized risk reduction and cognitive enhancement plan formulated, and clinical trial opportunities identified.

## Data Availability

Data sharing is not applicable to this article as no datasets were generated or analyzed during the current study.

## Abbreviations

AD: Alzheimer’s disease
ANU-ADRI: Australian National University Alzheimer’s Disease Risk Index
*APOE*: Apolipoprotein E
AUC: Area under the curve
BDSI: Brief Dementia Screening Indicator
BHS: Brain health services
CAIDE: Cardiovascular Risk Factors, Aging and Dementia
CHS: Cardiovascular Health Study
CSF: Cerebrospinal fluid
DSDRS: Diabetes-Specific Dementia Risk Score
FDG: 18-Fluorodeoxyglucose
FHS: Framingham Heart Study
HRS: Health and Retirement Study
MRI: Magnetic resonance imaging
Nfl: Neurofilament light
PET: Positron emission tomography
P-tau: Phosphorylated tau
SALSA: Sacramento Area Latino Study on Aging
TBI: Traumatic brain injury
